# Phonological and orthographic processing in basic literacy adults and dyslexic children

**DOI:** 10.1007/s11145-022-10347-6

**Published:** 2022-09-15

**Authors:** Régine Kolinsky, Méghane Tossonian

**Affiliations:** 1 Unité de Recherche en Neurosciences Cognitives (Unescog), Université Libre de Bruxelles (ULB), CP 191, 50 Ave. F. Roosevelt, 1050 Brussels, Belgium; 2 Fonds de La Recherche Scientifique-FNRS (FRS-FNRS), Brussels, Belgium

**Keywords:** Adult literacy, Dyslexia, Reading acquisition, Phonological processing, Orthographic processing

## Abstract

The aim of the present study was to examine the hypothesis that, compared to typically reading children matched on regular word reading, adults with basic literacy (either adult literacy students or adult basic education students) struggle on phonologically demanding tasks but are relatively performant on orthographic demanding tasks, and hence present a performance pattern similar to that of dyslexic children. Using various reading and phoneme awareness tests, we therefore compared the adults to both typically reading children from Grades 3 and 4 and dyslexic children, these two groups being matched to the adults on regular word reading. The dyslexic children were also compared to either chronological age- or reading level-matched children. The hypothesis was only partly supported by the data, as results depended on the subgroup of adults considered. While the literacy students presented poorer phoneme awareness and a somewhat stronger length effect in reading than the dyslexic children, the basic education students outperformed the latter on irregular word reading. The adults, and in particular the literacy students, also relied frequently on orthography in a complex phoneme awareness task. Taken together, these results suggest that adults with basic literacy rely more on visual memory than both dyslexic and typically reading children. This opens the question of whether the peculiar profile of these adults is intrinsic to adult literacy acquisition or is related to the way they are taught and trained to read and write. The results also highlight the need for better characterization of subgroups of adults with basic literacy.

## Introduction

The present study examined typically reading French-speaking Belgian children, dyslexic children, and adults who have had little schooling and hence display poor literacy*.* More specifically, in several French-speaking Belgian institutions, adults who have basic literacy skills are categorized either as adult *basic education students* or as adult *literacy students*. The latter complain primarily about their very low literacy level and therefore focus on literacy acquisition, while basic education students aim to review or upgrade basic concepts to obtain the basic education certificate usually issued at the end of elementary school. Given that not all countries organize distinct adult literacy and adult basic education classes, these participants will be called *basic literacy adults* (BL adults).

Several studies have reported that, compared to typical primary school children matched on reading level and/or ability to decode regular words, BL adults perform less well on phonologically demanding tasks such as reading or spelling pseudowords (in English: Greenberg et al., 1997; Thompkins & Binder, 2003; in French: Eme, 2006; Eme et al., 2014). Yet BL adults are relatively better on tasks involving orthographic processing, such as reading irregularly spelled words, at least in English (Greenberg et al., 1997; Thompkins & Binder, 2003; as far as we know, no study focused on irregular word reading in French-speaking BL adults).

The qualitative analysis of reading errors also suggested that BL adults rely less on phonology and more on orthographic processes than primary school children matched on general reading level (Greenberg et al., 2002). Such a strategy is displayed for instance in reading *sight words*, which are atypically spelled words that may be called *irregular* because they do not follow the basic correspondences between graphemes (letter or groups of letters) and phonemes (henceforth, GPCs), such as the English words <ocean> , <busy> , and <island> . In sight word reading, Greenberg et al. (2002) showed that BL adults misread more sight words as other real words than children, who instead produced more nonwords corresponding to acceptable decoding errors that follow the basic GPC rules (e.g., stating “deef” for the word <deaf>).

In addition, Binder and Borecki (2008) compared BL adults to skilled adult readers on a homophone silent reading task in which participants read short paragraphs for comprehension. The texts contained a contextually correct homophone (e.g., <break>), an incorrect homophone (e.g., <brake>), or a spelling control (e.g., <bread>), with the homophones being either visually alike, as <break> – <brake>, or more dissimilar, differing in at least their first letter, as <serial> – <cereal> . In agreement with past research (e.g., Rayner et al., 1998), skilled readers presented with short sentences showed no reading time differences between the correct homophone and the incorrect homophone (i.e., the wrong spelling in the sentence context, although the phonological code was correct, as when <brake> was present in the text although <break> was appropriate), provided the two homophones were visually similar. Thus, the phonological code was sufficient to activate the word’s meaning in skilled readers, who did not seem to notice that the wrong word was present. The BL adults were *better* at noticing the incorrect homophone, spending more time reading it compared to the correct homophone, although they presented a significant difference between the incorrect homophone condition and the spelling control condition, as skilled readers did, suggesting that both use phonological codes during word recognition.

The phonological limitations of BL adults are also illustrated by their striking difficulty in performing phoneme awareness tasks (e.g., Eme et al., 2014; Greenberg et al., 1997; Thompkins & Binder, 2003). Developing explicit, *metaphonological*, representations of the speech “unit(s)” denoted by the script is crucial for beginning readers. More specifically, reading in an alphabetic script is contingent on the grasping of the *alphabetic principle*, namely of the intuition that letters, alone or in combination (*graphemes*), stand for phonemes. Accordingly, phoneme awareness develops hand in hand with the acquisition of this principle (e.g., Morais et al., 1987a, b). Contrary to syllables, phonemes do not correspond to stable physical segments of speech (Liberman et al., 1967) and some (especially plosives) are unpronounceable in isolation (for further discussion on phonemes, see Morais, 2021). Hence, contrary to syllable awareness, the ability to segment speech into phonemes (e.g., as in a counting task) and to manipulate phonemes (e.g., in phoneme inversion, deletion, or addition tasks) depends crucially on alphabetic literacy. This is demonstrated by the fact that phoneme awareness is virtually absent in both prereading children (e.g., Liberman et al., 1974) and alphabetic illiterate adults (Morais et al., 1979, 1986; Read et al., 1986), especially for phonemes that are not pronounceable in isolation (Morais et al., 1986). In contrast, these individuals demonstrate relatively good syllable awareness (e.g., Liberman et al., 1974; Morais et al., 1986).

BL adults perform much worse than reading-level matched children on phoneme awareness tasks such as phoneme counting (Read & Ruyter, 1985), deletion (e.g., “say smile again but without s”, Eme, 2006; Eme et al., 2014; Greenberg et al., 1997), addition (Read & Ruyter, 1985) or reversal (e.g., “but” become “tub”, Eme, 2006; Eme et al., 2014). In Greenberg et al. (1997), for instance, BL adults’ average phoneme deletion performance was about half of the children’s performance (32 vs. 60% correct, respectively). In Read and Ruyter (1985), they succeeded on phoneme counting at about 39%,^1^on the average, although they were quite good at counting syllables (almost 77%, on the average). BL adults seem to have special difficulty with the segmentation of consonant clusters, as when required to say what the French word /frit/ would become if the initial phoneme were removed (Eme et al., 2014). In the same way as in reading and spelling tasks, BL adults rely strongly on orthographic cues to perform phonological awareness tasks. For instance, when required to circle pairs of written words that rhyme in speech, they frequently overlook the differently spelled rhymes (e.g., the English words <fuel> and <mule>), performing at chance level in this condition (Greenberg et al., 1997, 2002).

Taken together, all these results point to a relative strength of orthographic codes compared to a relative weakness in the use of phonology in BL adults. As they hardly use phonological decoding, BL adults seem to turn to alternative strategies relying on orthographic knowledge and spelling rules, as well as on memory of specific words (e.g., Greenberg et al., 1997, 2002; Thompkins & Binder, 2003).

Phonological abilities are also seriously compromised in children presenting developmental dyslexia (e.g., Rack et al., 1992), a severe and persistent difficulty in learning to read and write despite adequate intelligence, sensory and cognitive abilities, and educational opportunities (Snowling, 2000). Specifically, one of the most important correlates of developmental dyslexia is the inability to segment speech into phonemes (see discussions in e.g., Puolakanaho et al., 2007; Ramus et al., 2003; Snowling & Melby-Lervåg, 2016). For this reason, it was suggested that the BL adults’ reading-writing profile resembles profiles usually associated with younger learning-disabled readers, namely children with developmental dyslexia (e.g., Greenberg et al., 1997). Yet, as discussed by Greenberg et al. (1997), a phonological deficit may reflect either a fundamental dysfunction or inadequate instruction. Unfortunately, very few studies on phonological abilities compared BL adults to children presenting developmental dyslexia. To our knowledge, the only study that included such a comparison focused only on metaphonological abilities and was run on readers of a very consistent orthographic code (Spanish), in which graphemes map relatively consistently on phonemes, and vice-versa (Jiménez et al., 2010).

In former studies, the comparison of BL adults to typical readers is also hindered, but now by the fact that most of those studies were run on English, a highly inconsistent code. Indeed, in English numerous graphemes can have multiple pronunciations and numerous phonemes can be spelled in more than one way, making it an “*outlier* orthography in terms of spelling-sound correspondence” (Share, 2008, p. 584). This peculiarity gives rise to important differences between reading and spelling acquisition in English and other languages. As a matter of fact, the consistency of the orthographic code impacts reading acquisition both quantitatively and qualitatively, with slower acquisition (e.g., Seymour et al., 2003) and stronger reliance on larger units (e.g., rimes, Ziegler et al., 2001) in inconsistent than consistent orthographies.

Only a few studies investigated BL adults’ reading and writing skills in the French language (e.g., Eme, 2006; Eme et al., 2014), which is peculiar as regards consistency. Although French is often referred to as presenting a very difficult orthographic code, it presents much more inconsistencies in spelling than in reading, with numerous phonemes that can have multiple spellings. This inconsistency of the phoneme-grapheme correspondences (PGC) allows only about 21% of French monosyllabic words and 28% of English monosyllabic words to be spelled correctly (Ziegler et al., 1996). Yet, whereas English is exceptionally inconsistent in both spelling and reading, in French the grapheme-phoneme correspondences (GPC) used to read words are more consistent than usually thought (Content & Peereman, 1992), in fact much more consistent than in English. This makes it possible to correctly read about 88% of French monosyllabic words on the basis of GPCs (Ziegler et al., 1996) vs. only 69% in English (Ziegler et al., 1997). Consistently, at the end of Grade 1 French children perform far better than English children (about 75 vs. 40%, respectively, Seymour et al., 2003), but worse than children from other, more consistent, European orthographies, who display word reading performance of about 95%.

The present study aimed at examining the hypothesis that in contrast to typically developing children, French-speaking BL adults and dyslexic children will show a similar pattern of weakness in phonology and relative strength in orthography. It adds to previous evidence in two ways. First, to our knowledge, up to now no study examined in French whether BL adults compared to reading-age matched typical children rely more on orthography in both reading (as attested in English by better irregular word reading in BL adults than reading-age matched children, Greenberg et al., 1997; Thompkins & Binder, 2003) and complex metaphonological tasks (Greenberg et al., 1997, 2002). Second, contrary to previous studies, we compared the BL adults to both dyslexic children and typical children.

As in former studies, we adopted a reading level match design. As Greenberg et al., (1997, 2002), we compared BL adults to children matched on reading regular words of varying difficulty. In the present study, in agreement with the institution teachers’ intuitions, the BL adults presented a reading level below the one usually observed at the end of primary school, being almost indistinguishable from children from Grades 3 and 4 (henceforth, G3–4) on reading the whole set of regular words of a standardized reading French test battery (*Batterie d’évaluation du langage écrit*—BELEC, Mousty et al., 1994). These G3-4 children were thus considered as reading-level (RL) controls for the BL participants. In addition to these participants, typically reading children from Grades 1 and 2 (henceforth, G1–2) and from Grades 5 and 6 (henceforth, G5–6) were included as RL and chronological age-level (CA) matched controls for the dyslexic group, respectively. The children of the RL control group had indeed been selected to be matched to the dyslexic children on regular word reading. Finally, we also directly compared a subgroup of the dyslexic children to a subgroup of the BL adults matched on regular word reading. Therefore, to align with the reading level match design, comparisons were made on pairs or triplets of groups rather than through a single omnibus analysis that would include all study groups but not control for reading level.

Participants were presented with several other reading materials as well as with metaphonological tasks. The most phonologically demanding tasks were pseudoword reading, phoneme deletion, and auditory acronym. On the contrary, irregular word reading aimed at tapping orthographic processes, as mere coding or decoding abilities are insufficient to read or spell such words. In addition, the material of the auditory acronym task was designed to disclose possible reliance on orthography. Participants had to isolate the first phonemes of two spoken words and blend them to form a new word, for instance to answer /gɑ̃/ (“gant”, meaning “glove”) to the pair /gʁav ɑ̃tɔʁs/ (“grave entorse”, meaning “severe sprain”). Relying on the orthographic representation of the words to perform the task would lead to an incorrect response, namely to the word /ʒə/ (“je”, meaning “I”) in the above example, as the letter <g> is pronounced /ʒ/ before <e>.

Based on our hypothesis, we predicted that, compared to typical children reading regular words at the same level, the BL adults would present lower performance on phonologically demanding tasks such as pseudoword reading, phoneme deletion and auditory acronyms, but better performance on orthographic demanding tasks such as irregular word reading. The BL adults were also expected to rely more on orthography when the task taps complex phonological abilities, as the auditory acronym task. No difference was expected between the BL adults and the dyslexic children matched on regular word reading.

To examine these predictions, we analyzed reading performance not only as a function of regularity (regular vs. irregular words) but also in terms of lexicality (pseudowords vs. words), as pseudoword reading is more phonologically demanding than word reading.

In addition, we also looked at the effect of word length on reading performance. As a matter of fact, beginning readers’ reading performance is closely related to the number of letters (or other sublexical units) in a word: as word length increases, their reading performance tends to decrease and their reading latencies to increase. This so-called length effect is believed to be a marker of a sublexical serial (letter-by-letter) decoding. Typical reading acquisition is characterized by a decrease in word-length effects, with weaker effects for more advanced readers than for beginning readers (e.g., in English: Samuels et al., 1978; in French: Mousty & Leybaert, 1999), whereas persistently elevated word-length effects are characteristic of developmental dyslexia (e.g., in English: Landerl et al, 1997; Ziegler et al., 2003; in French: Juphard et al., 2004). The decrease in the effect is often considered to reflect a gradual shift from sublexical serial reading to parallel processing of letter strings (e.g., Coltheart et al., 2001; Ziegler et al., 2003) and/or reliance on a lexical strategy of reading whole words. Data collected on second-grade Dutch children by van den Boer et al. (2013) support the idea that the length effect taps serial reading, as it is negatively correlated with phoneme deletion performance (van den Boer et al., 2013). According to these researchers, phoneme awareness might be related to the length effect through its relation with phonological recoding, namely, “print-to-sound” translation. Successful phonological recoding requires basic awareness of phonemes and, according to the *self-teaching hypothesis* (Share, 1995, 1999), is itself crucial for building and strengthening orthographic representations. Hence, poor phonological awareness results in poor elaboration of orthographic representations, and therefore in persistent reliance on serial processing and sensitivity to item length. Individual differences in the size of the length effect might thus be considered as an indication of the development of the reading system. Yet, to our knowledge, the length effect has not been investigated so far in BL adults.

## Method

### Participants

All the BL adult participants were students involved in adult basic education or literacy classes organized by an institution of the French Community of Belgium aimed at unemployed jobseekers with few qualifications and little schooling. Basic education students were French speaking people who had not completed their basic or primary education and hence aimed at revising or upgrading basic notions (e.g., in history, geography, French and math) to obtain the certificate of basic education usually delivered at the end of primary school in Belgium. Students of literacy classes were also French speaking people with little schooling but complained mainly about their very low literacy level; hence they specifically focused on reading and writing as well as math acquisition. One participant had to be discarded because she was not fluent in French. The final sample included 25 BL adults (17 adult basic education students and 8 adult literacy students), mostly women (56%), with a mean age of 34.9 years (from 19 to 55 years, SD = 11.05). Most (68%) were native French speakers. The others were also quite fluent in French, as most of them (83%) come either from multilingual countries where French is an official language (e.g., Belgium; Democratic Republic of the Congo) or from countries where French remains used in media and education (from primary school), due to colonial history (e.g., Algeria). In addition, a majority (67%) of the non-native French speakers had lived in Belgium for over 15 years. They were largely unemployed (80%) after having completed vocational studies (≃ 70%); yet some (26%) only completed primary education. Most (83%) had attended school in Belgium. According to the institution teachers, most of these BL adults presented a reading level below the one usually observed at the end of primary school.

The typically reading children were recruited in five different public schools of the French Community of Belgium. The initial G1–2 sample included 45 children (28 1st Graders). Among them, 26 (9 1st Graders) were reading the whole set of regular words at the same level as the dyslexic children (see details in the Results section) and were thus included as RL controls for the dyslexic group. They were aged between 6.2 and 8.8 years (average: 92.23 months, SD = 9.09). The 47 G5–6 children were aged between 91/2 and 11 years (average: 124.38 months, SD = 4.72). On the average, they were roughly of the same age as the dyslexic children (see below), *BF*_01_ = 0.52, and were thus considered as CA controls for the dyslexic group. There were also 41 G3–4 typically reading children aged between 8 and 101/2 years (average: 103.45 months, SD = 9.61). As detailed in the Result section, they were matched to the BL adults on reading the whole set of regular words and hence were considered as RL controls for the BL group.

The dyslexic children were recruited either from four different schools of the French Community of Belgium for children with learning disabilities, or via their language therapist. All had been previously diagnosed with developmental dyslexia, had normal IQ, and did not have auditory or visual impairment. In addition, no psychological or neurological deficits was reported. All benefited from remediation sessions. They were 53 children aged between 8 and 12 years (average: 126.92 months, SD = 11.31). To check for these children’s particular reading profile, we performed a deviance analysis in the same way as Ramus et al. (2003), considering their performance on irregular words and on pseudowords. For each one of these two variables, children’s performance was transformed into a standardized *z*-scores calculated by reference to the mean and standard deviation of the CA controls, after removing the CA participants with extremely low performance (*z* ≤ − 1.65, relative to the distribution of their own group). We then examined the proportion of dyslexic children scoring significantly below this criterion. Any *z*-score ≤ − 1.65 (i.e., corresponding to or below the 5th percentile of a normal distribution) relative to the CA controls was considered as reflecting deviant performance. The large majority (49 children) presented deviant performance on both irregular words and pseudowords; one child presented deviant performance only on pseudowords, and three children presented deviant performance only on irregular words.

### Materials, procedure and scoring

All the tests were presented individually. Participants were examined in a quiet room at either their literacy/adult basic education institution (adults) or school (children).

#### Reading tests

Two reading subtests taken from a standard French test battery, the BELEC (Mousty et al., 1994), were administered to all participants (25 BL adults, 53 dyslexic children, 26 G1–2 children, 41 G3–4 children, and 47 G5–6 children). Both tests consist of reading aloud the items, these being blocked by condition. The *Mécanismes d’Identification de Mots* (MIM) subtest includes 72 items, 48 words (half frequent, e.g., “image”, “croix”, half rare, e.g., “rival”, “pieux”) and 24 pseudo-words, half of all items being short ones (5 letters, as in the former examples), the others long ones (9–12 letters, e.g., “satisfaction”, “catastrophe”). Six words and six pseudowords served as training trials, presented just before the corresponding condition. We administered only one of the two series originally constructed (the “A” series). The *Regularité* (REGUL) subtest includes 24 regular words and 24 irregular words. Irregular words are either exceptions to the GPCs (e.g., “oignon”) or words in which a grapheme has an unusual pronunciation (e.g., “mille”). These words are matched on number of letters, phonemes, and syllables, and approximately on frequency. For both tests, the number of accurately read items was registered for each condition of lexicality, item length (in MIM) and orthographic regularity (in REGUL).

Reading performance on the 48 words of the MIM subtest and on the 24 regular words of the REGUL subtest was aimed at matching the G3–4 typically reading children to the BL adults as well as the dyslexic children to their RL controls.

#### Phonological awareness

We presented the participants with two deletion tasks of the BELEC (Mousty et al., 1994), using the original tape-recorded stimuli. The first task was syllable deletion (16 items with a consonant–vowel-consonant -vowel –CVCV– structure, e.g., /vymo/). It was used to have a control of the understanding of the task. As it led to ceiling or near-to ceiling performance in several groups, in particular in the G5-6 children, who all scored at 100% correct, we did not include this test in further analyses (average correct scores in the other groups: 99.27% in the G1–2 children, SD = 2.08; 98.66% in the G3-4 children, SD = 4.29; 94.87% in the dyslexic children SD = 11.29; and 91.41% in the BL adults, SD = 5.04). The second task was initial phoneme deletion, which included 16 CVC items with simple onsets, (e.g., /seg/) and 10 CCV items with complex onsets (e.g., /klo/). This manipulation of syllable structure was made because previous empirical evidence has shown that linguistic complexity impacts phonological awareness performance (e.g., Sthal & Murray, 1994). In both tasks, participants were asked to delete the first “sound” of a pseudoword and produce the resulting pseudoword. Four training trials in which corrective feedback was provided were presented before each material.

The auditory acronym task of the BELEC was also presented. It consisted of four training trials with corrective feedback and 16 experimental trials on which participants had to isolate the first phonemes of two auditorily presented words and blend them to form a new word. As already illustrated, on each pair there were (at least) two possible word responses, the correct phonology-based response, and an incorrect orthography-based responses. We registered the number of responses of each type.

The syllable and phoneme deletion tests were presented to 16 BL adults, 28 dyslexic children, 17 G1–2 children, 28 G3–4 children and 36 G5–6 children. Auditory acronyms were not presented to the G3–4 children but to 16 BL adults, 22 dyslexic children, 17 G1–2 children and 26 G5–6 children.^2^

### Data analysis

As the BL adult participants were not very numerous given the large variation in performance usually reported in this population and as some tests aimed at checking whether there was no group difference, namely at testing the null hypothesis (H_0_: no group difference) against the alternative one (H_1_: there is a group difference), all data were analyzed through Bayesian analyses, including Bayesian ANOVAs, using the opensource graphical statistical package JASP (Version 0.15; https://jasp-stats.org/). Except when stated otherwise, all Bayesian analyses used the default priors proposed in JASP: a zero-centered Cauchy prior distribution with r scale parameter ≈ 0.707 for post hoc t tests and r scale fixed effects = 0.50 for ANOVAs.

In those analyses, the Bayes factor *BF*_10_ indicates how likely the data are under H_1_ compared with H_0_ and are directly interpretable as an odds ratio. A Bayes factor of 1 means that the data are equally likely to occur under H_0_ and H_1._ A value greater than 1 indicates that the data are more likely to have occurred under H_1_ than under H_0_, and vice versa when the Bayes factor is below 1. Jeffreys (1961) proposed a set of verbal labels to categorize different Bayes factors according to their evidential impact; for instance, a value of 3, which indicates that the data are three times more likely under H1 than under H_0_, is considered as substantial evidence for H_1_ over H_0_; odds greater than 10 are considered as strong evidence (Jeffreys, 1961; see also Raftery, 1995). Conversely, a Bayes factor of 0.33 (or lower) indicates substantial support for H_0_, namely that the data are about three times more likely to occur under H_0_ than under H_1_ (1/0.33 = 3.03), which is referred to as *BF*_01_ (= 1/BF_10_).

For the ANOVAs, in addition to model comparisons with the null model, we estimated the contribution of effects and interactions using the JASP matched-model comparison procedure suggested by Mathôt (2017). This procedure compares models that contain the effect with equivalent models stripped of the effect. Thus, higher-order interactions are excluded from simple effects. Concerning interactions, this allows evidence for the interaction to be evaluated on its own by comparing the *BF*_10_ of a model with the interaction against the *BF*_10_ of a model with only the main effects (i.e., without the interaction). For instance, if there are two factors, Lexicality and Group, the Lexicality X Group *BF*_inclusion_^3^ corresponds to the *BF*_10_ value for the model [(Lexicality + Group) + (Lexicality X Group)] divided by the *BF*_10_ value for the model [Lexicality + Group].

### Ethics statement

Ethics approval for the study was granted by the Faculté des Sciences Psychologiques et de l’Éducation, Université Libre de Bruxelles (protocol no. 159/2019). Written informed consent was obtained from the BL adults. Yet, to help them in reading the text, we also presented them it orally. For children, informed consent was obtained from their parents, and oral agreement was obtained from each child.

## Results

For each of the five sets of comparisons presented below in five separate subsections, we began by examining performance on the whole set of 72 regular words of varying difficulty of the BELEC (see Method section). This allowed checking whether regular word reading was roughly equivalent between the BL adults and the G3–4 children, between the dyslexic children and the G1–2 controls, as well as between the BL adults and the dyslexic children.

Then, in separate Bayesian ANOVAs (repeated measures analyses for all tasks, expect for the comparison between three groups of children on auditory acronyms), we contrasted reading performance on the 24 regular and 24 irregular words that were matched on length and frequency (REGUL subtest of the BELEC) as well as on the 48 words and 24 pseudowords that were matched on length (MIM subtest of the BELEC). We further checked whether item length modulated reading performance by comparing short to long items,^4^and examined metaphonological performance (syllable deletion, phoneme deletion, and auditory acronyms).

For the ANOVAs, we will often numerically report only those effects or interactions for which there was substantial or close to substantial evidence (Bayesian Factor ≥ 3 for evidence supporting H_1_ and ≤ 0.33 for H_0_). More detailed results are presented in Appendices 1–5 for each of the five sets of the between-groups comparisons presented in the following five subsections, respectively. Appendix 6 presents some finer-grained results and analyses ran only on the BL adults.

### Comparisons between the reading-level matched basic literacy adults and typically reading children

We first checked whether the BL adults and the typically reading G3–4 children were matched in reading level on the whole set of regular words. Table 1 shows that the BL adults were almost undistinguishable from the G3–4 typical readers on this material. The data were about three times more likely under H_0_ than under H_1_, *BF*_01_ = 3.53, which is considered as substantial evidence for the idea that there is no group difference on this material. Thus, the G3–4 children can be considered as RL controls for the BL adult group.

**Table 1:** Average reading scores (in %) observed in each group on the whole set of regular words

	BL adults	G3–4	Dyslexic children	G1–2	G5–6
N	25	41	53	26	47
Mean	93.78	93.12	79.04	79.11	97.99
SD	[6.97]	[4.48]	[13.26]	[18.21]	[1.94]
Range	75–100	80.56–100	37.5–97.22	33.33–95.83	93.07–100

Standard deviations in brackets. BL: Basic literacy adults; G1–2, G3–4, G5–6: children from Grades 1 and 2, 3 and 4, or 5 and 6, respectively

**Fig. 1:**
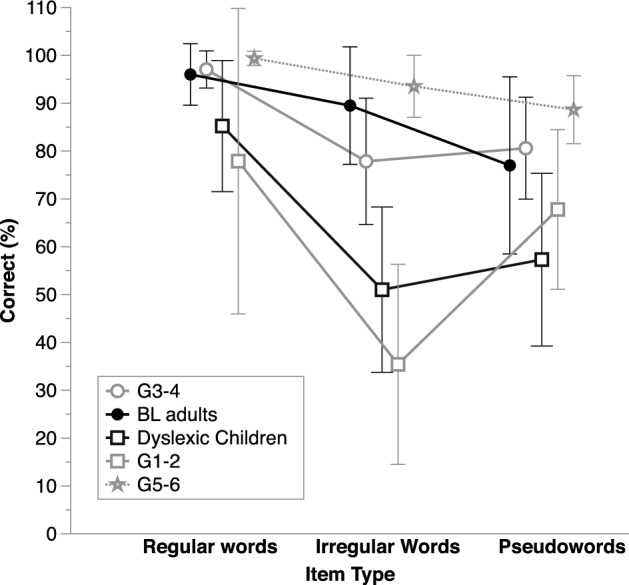
Average correct performance (%) on the reading tests, separately for each item type and in each group of participants (BL adults: Basic literacy adults; G1–2, G3–4, G5–6: children from Grades 1 and 2, 3 and 4, or 5 and 6, respectively). Error bars show standard deviations

In the ANOVA that considered Regularity (regular vs. irregular words) in addition to Group (BL adults vs. G3-4 children), there was very strong evidence for a Regularity X Group interaction, *BF*_inclusion_ = 718.17, in addition to evidence for the main effects of Regularity and of Group (Fig. 1). There was some evidence that groups did not differ on regular words, *BF*_01_ = 2.86, and strong evidence that, on the contrary, the BL adults read irregular words better than the G3–4 typically reading children, *BF*_10_ = 42.72.

The ANOVA that considered Lexicality (words vs. pseudowords) in addition to Group showed very strong evidence for a main effect of Lexicality, with better performance on words than pseudowords, *BF*_inclusion_ = 2.65E+11 (Fig. 1). In addition, there was some evidence that Group did not affect performance, *BF*_exclusion_ = 2.95.^5^ In the ANOVA that included Group and Length (short vs. long items; see average values in Appendix 1), there was only evidence for a main effect of Length, *BF*_inclusion_ > 120 000, with better performance on short items (on average, 90.4%, SD = 7.6) than on long ones (on average, 84.72%, SD = 10.54).

One-tailed Kendall rank correlation coefficients showed very strong evidence for a negative correlation between the size of the length effect and phoneme awareness, as estimated by calculating the average performance on phoneme deletion and auditory acronyms, *τ-b* = − 0.57, *BF*_*−*0_ = 126.81, and very strong evidence for a positive correlation with the proportion of orthographic errors made on auditory acronyms, *τ-b* = 0.57, *BF*_+0_ = 47.32 (the latter being observed only in the BL adults). These results agree with former data collected on second-grade Dutch children (van den Boer et al., 2013) and support the notion that the length effect taps serial reading.

For the BL adults, we registered separately the data on short and long words vs. pseudowords (see footnote 4) and hence ran a separate ANOVA including both Lexicality and Length as factors. There was very strong evidence for a Lexicality X Length interaction, *BF*_inclusion_ = 602.25, in addition to the main effects of Lexicality, *BF*_inclusion_ = 4.21E+6, and Length, *BF*_inclusion_ = 1465.34. The interaction reflected the fact that the length effect was significant only on pseudowords, *BF*_01_ = 3398.1, on which it reached 20% (average correct: 67% on long pseudowords, SD = 24.35, vs. 87% on short ones, SD = 15.61). On words, the length effect was barely noticeable, *BF*_01_ = 0.51 (average correct: 91.83% on long words, SD = 8.29, vs. 93.5% on short ones, SD = 8.17). Yet, this effect is often evaluated by considering reading latencies, which is a more sensitive index than reading accuracy. Although we did not collect reading latencies per se, on the BL adults we recorded the total reading times separately for each type of word and pseudoword (see average values in Table 2). The ANOVA ran on these scores showed that, as on reading accuracy, there was very strong evidence for a Lexicality X Length interaction, *BF*_inclusion_ = 2.04E+9, in addition to the main effects of Lexicality, *BF*_inclusion_ = 1.04E+8, and Length, *BF*_inclusion_ = 2.3E+15. Although this interaction reflects a stronger length effect on pseudowords than on words (on average, 7.74 vs. 2.43 s, respectively), there was very strong evidence for this effect on both words, *BF*_01_ = 26458.4, and pseudowords, *BF*_01_ = 3.52E+11. This pattern corresponds to what is usually found with adult skilled readers, where length effects are typically stronger for pseudowords than for words (e.g., Weekes, 1997).

**Table 2:** Average total reading times and inverse efficiency scores (in s) observed in the basic literacy adults, for each item type

	Total reading times				Inverse efficiency scores			
	Words		Pseudowords		Words		Pseudowords	
	Short	Long	Short	Long	Short	Long	Short	Long
Mean	4.72	7.15	5.4	13.14	5.17	8.02	6.91	810.49
SD	[2.33]	[2.42]	[2.2]	[3.78]	[2.79]	[3.33]	[4.92]	[3922.96]
Range	1.97–13.35	3.55–11.31	2.11–10.79	7.91–23.39	1.97–15.25	3.71–15.08	2.11–25.91	10.55–19640

Standard deviations in brackets

For examining correlations, we combined the BL adults’ reading times with their accuracy data by using inverse efficiency scores (IES, Townsend & Ashby, 1978; see Table 2).^6^ There was substantial  evidence for a negative correlation between the size of the word length effect and phoneme awareness, *τ-b* = − 0.50, *BF*_−0_ = 19.52, and some evidence for a negative correlation between the pseudoword length effect and phoneme awareness, *τ-b* = − 0.44, *BF*_−0_ = 8.29. There was also strong evidence for a positive correlation between the size of the word length effect and the proportion of orthographic errors made on auditory acronyms, *τ-b* = 0.60, *BF*_+0_ = 83.41, and some evidence for a similar relation between the size of the pseudoword length effect and the proportion of these errors, *τ-b* = 0.42, *BF*_+0_ = 6.79.

**Fig. 2:**
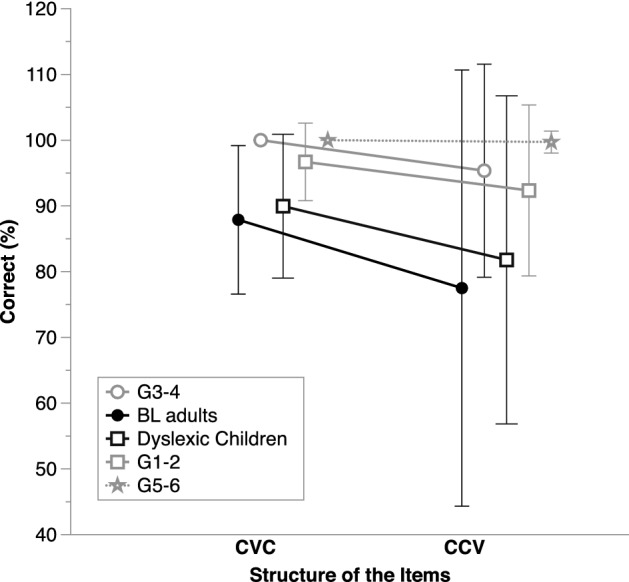
Average correct performance (%) on phoneme deletion, separately for each structure (simple onset: CVC items; complex onsets: CCV items) and in each group of participants (BL adults: Basic literacy adults; G1–2, G3–4, G5–6: children from Grades 1 and 2, 3 and 4, or 5 and 6, respectively). Error bars show standard deviations

As illustrated in Fig. 2, the BL adults displayed poorer phoneme awareness than the G3–4 typical readers. This was confirmed by an ANOVA on phoneme deletion scores, which showed strong evidence for a Group effect, *BF*_inclusion_ = 26.4. Yet, contrary to what had been reported in former work (Eme et al., 2014), there was no convincing evidence for an effect of Structure (items with simple onsets—CVC– vs. items with complex onsets –CCV), nor for an interaction between Structure and Group. For both CVC and CCV items, G3-4 children outperformed BL adults by more than 12%.

### Comparisons between the dyslexic children and reading-level or chronological age matched typically reading children

As can be seen in Table 1, on the whole set of regular words, the dyslexic children strongly differed from the CA controls (G5–6 typically reading children), *BF*_10_ = 8.25E+12, but were almost undistinguishable from the G1-2 typical readers, *BF*_01_ = 4.06. Thus, the G1–2 children can be considered as RL controls for the dyslexic group.

The ANOVA that considered Regularity and Group (dyslexic children, G1–2, G5–6) as factors showed very strong evidence for a Group by Regularity interaction, *BF*_inclusion_ = 4.29E+19, in addition to the main effects of Regularity and of Group (Fig. 1). Compared to their reading-level matched controls, there was no conclusive evidence that dyslexic children differed on regular words, *BF*_10_ = 0.59, but there was very strong evidence that they were better at reading irregular words, *BF*_10_ = 39.04, although they underscored the G5–6, *BF*_10_ = 2.59E+25, as they did for regular words, *BF*_10_ = 3.12E+7.

The ANOVA that considered Lexicality and Group as factors showed very strong evidence for a Lexicality X Group interaction, *BF*_inclusion_ > 1900, in addition to the main effects of Regularity and of Group. As illustrated in Fig. 1, on pseudowords dyslexic children performed worse than both the RL and the CA controls, *BF*_10_ = 3.28 and = 8.99E+15, respectively, whereas on words they only underperformed the CA controls, *BF*_10_ = 4.67E+13, not the RL controls, *BF*_10_ = 0.41. In the ANOVA that considered Length in addition to Group (see average values in Appendix 2), there was evidence for the main effects of Group, *BF*_inclusion_ = 3E+15, and of Length, *BF*_inclusion_ = 1.93E+8, with better performance on short items (on average, 83.75%, SD = 14.44) than on long ones (on average, 76.59%, SD = 19.38). Again, the size of the length effect tended to correlate negatively with performance on phoneme deletion, *τ-b* = − 0.17, *BF*_−0_ = 5.17, and auditory acronyms, *τ-b* = − 0.20, *BF*_−0_ = 4.9.

The dyslexic children displayed relatively poor phoneme awareness. There was very strong evidence for a Group effect in the ANOVA on phoneme deletion scores, *BF*_inclusion_ = 11752.86 (Fig. 2). Yet there was no convincing evidence for an effect of Structure, nor for an interaction between Structure and Group. Across CVC and CCV items, dyslexic children displayed lower scores than the CA controls, *BF*_10_ = 745970.56. They also tended to perform worse than the RL controls, although the evidence was weak, *BF*_10_ = 2.64. On auditory acronyms, the ANOVA showed evidence for a Group effect, *BF*_10_ = 21.67, with dyslexic children (on average, 82.67%, SD = 17.57) performing more poorly than the CA controls (96.88, SD = 5.08), *BF*_10_ = 92.29, but no evidence supporting a difference with the RL controls (on average, 90.07, SD = 15), *BF*_10_ = 0.67.

### Comparisons between the reading-level matched basic literacy adults and dyslexic children

On the full samples, the BL adults read the whole set of regular words far better than the dyslexic children, *BF*_10_ = 9157 (Table 1). We thus tried to check whether it was possible to match the reading performance of the BL adults and of the dyslexic children on the whole set of regular words. This was quite difficult because the average scores of many dyslexic children were lower than the minimum score observed in the BL adults. Yet, we succeeded in selecting 16 dyslexic children and 14 BL adults (half being adult literacy students) who did present similar regular word reading scores, with 89.41% (SD = 6.21) and 89.98% (SD = 7.33) average correct, respectively, *BF*_01_ = 2.84.

**Fig. 3:**
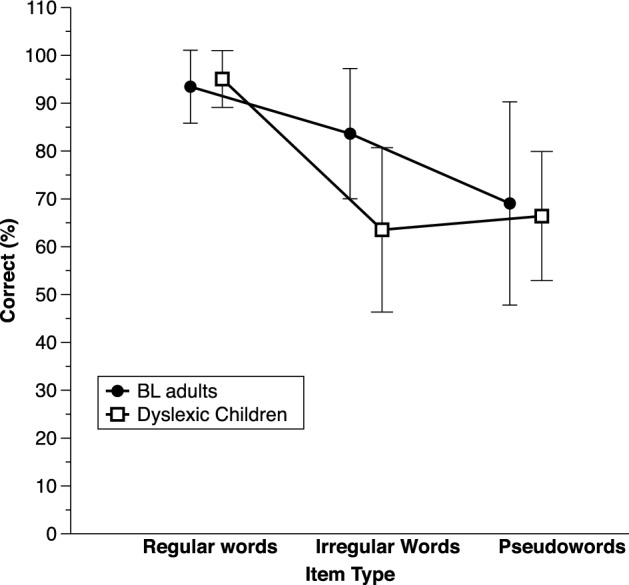
Average correct performance (%) on the reading tests, separately for each item type and for the basic literacy (BL) adults vs. dyslexic children matched on regular word reading. Error bars show standard deviations

Considering these two subgroups, in the Bayesian ANOVA considering Regularity and Group as factors there was very strong evidence for a main effect of Regularity, *BF*_inclusion_ = 2.83E+6, as well as for a Regularity X Group interaction, *BF*_inclusion_ > 240. As illustrated in Fig. 3, although reading the regular words at about the same level as the dyslexic children, *BF*_10_ = 0.4, the BL adults read the irregular words much better, *BF*_10_ = 21.95.

The ANOVA considering Lexicality and Group as factors showed very strong evidence for a main effect of Lexicality, *BF*_inclusion_ = 6.46E+6, with better performance on words than on pseudowords (Fig. 3). Evidence regarding the Group effect was unconclusive but there was substantial evidence that there was no Group X Lexicality interaction, *BF*_exclusion_ = 3.07. In the ANOVA taking Length in addition to Group, there was very strong evidence for a main effect of Length, *BF*_inclusion_ > 1000, without evidence for a Group by Length interaction (see average results in Appendix 3). Overall, performance was better on short items (on average, 84.72%, SD = 9.48) than on long ones (on average, 76.76%, SD = 17.77).

**Fig. 4:**
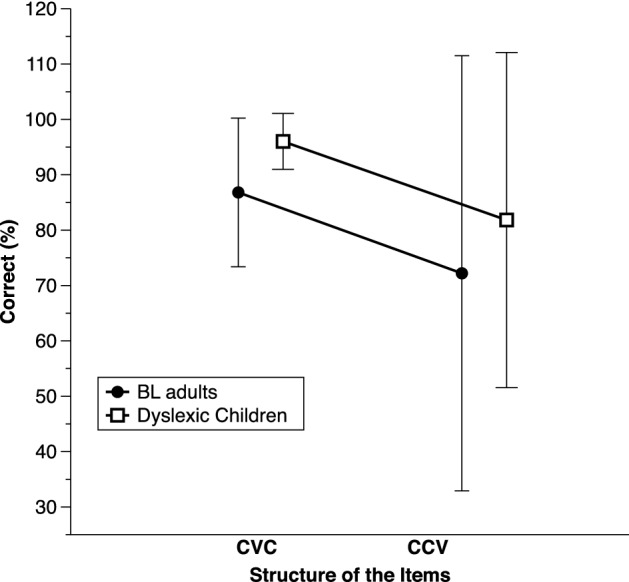
Average correct performance (%) on phoneme deletion, separately for each structure (simple onset: CVC items; complex onsets: CCV items) and for the basic literacy (BL) adults vs. dyslexic children matched on regular word reading. Error bars show standard deviations

Phoneme deletion was about 10% poorer in the BL adults compared to the dyslexic children on both CVC and CCV items (Fig. 4). Yet, there was evidence neither for main effects nor of a Group X Structure interaction in the ANOVA on deletion scores. On auditory acronyms, BL adults tended to perform more poorly than the dyslexic children, with average scores of 55.56% (SD = 27.68) and 83.33% (SD = 12.3), respectively. Yet, again, the effect was weak, *BF*_10_ = 2.06. In both phoneme awareness tasks, the weak Group effects probably reflects the fact that there were few participants and large variability on these tasks.

The BL adults’ poor score on auditory acronyms reflects the fact that most of them strongly resorted to orthography in performing this task, with an average proportion of orthographic errors of 16.02% (SD = 16.3) on the whole sample of BL adults and of 23.61% (SD = 18.16) on the subsample matched to the dyslexic children for regular word reading. Unfortunately, error types were unavailable for the dyslexic children. Yet it is interesting to note that the average proportion of orthographic errors was much lower (3.04%, SD = 0.88) in the 11 G5–6 typically reading children for whom this information was available.

We expected the proportion of orthographic errors to be negatively correlated with reading skills. To check for this idea, we considered a composite reading score computed by averaging performance across the various materials of the reading tests (MIM and REGUL). In the BL participants, the proportion of orthographic errors correlated negatively with this composite reading score, *τ-b* = − 0.50 according to a one-tailed Kendall rank correlation coefficient, *BF*_−0_ = 18.25.

### Are adult literacy students and adult basic education students similar on reading and phoneme awareness?

Up to now we considered the BL adults in a monolithic way, as all were French speaking people with little schooling and relatively low levels of literacy. Yet the BL adult group included both basic education students (17 participants) and students of literacy classes (8 participants), the latter complaining mainly about their very low literacy level. Running similar analyses to those used in the first three sets of comparisons, we checked whether reading and phoneme awareness were actually poorer in literacy students than in basic education students, and whether the comparison of each of these two BL adult subgroups with the G3–4 children would lead to similar results or not.

On the whole set of regular words, the G3–4 children displayed an average reading level (cf. Table 1: 93.12%, SD = 4.48) that was in-between that shown by the literacy students (88.72%, SD = 8.61) and that shown by the basic education students (96.16%, SD = 4.65). Consequently, the evidence for a Group effect in the ANOVA contrasting the G3-4 children to the two BL adult subgroups, *BF*_inclusion_ = 6.84, was only due to the difference between the two adult subgroups, *BF*_10_ = 5.32. None of these two subgroups differed from the G3-4 children, *BF*_10_ = 1.94 for the comparison with the literacy students and = 2.49 for the comparison with the basic education students. Thus, although both BL adult subgroups read the whole set of regular words at a level roughly comparable to that of the G3–G4 children, the literacy students read these words slightly less correctly than the basic education students.

**Fig. 5:**
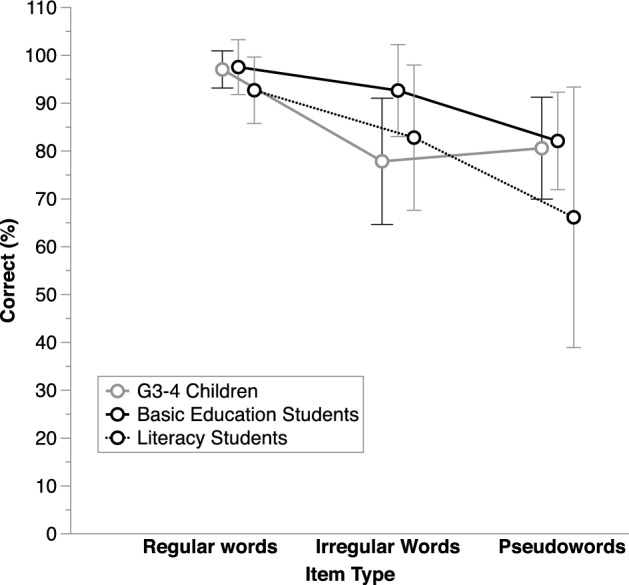
Average correct performance (%) on the reading tests, separately for each item type and in each subgroup of basic literacy adults (basic education vs. literacy students) as well as in the G3–4 (children from Grades 3 and 4) matched on regular word reading. Error bars show standard deviations

In the ANOVA on the regular and irregular words matched on length and frequency that contrasted the G3–4 children to the two BL adult subgroups (Fig. 5), there was very strong evidence for a Group X Regularity interaction, *BF*_inclusion_ = 526.13. Indeed, the difference between the literacy and basic education students was slightly more pronounced on irregular words than on regular ones. Hence, on irregular words only the basic education students, *BF*_10_ = 209.47, but not the literacy students, *BF*_10_ = 0.5, displayed better performance than the G3–4 children. The performance drop on irregular words, namely the regularity effect, was thus less marked in basic education students (on the average, 4.9%, SD = 7.4) than in the two other groups (on the average, 9.9%, SD = 15.42, in the literacy students and 19.21%, SD = 11.89, in the G3–4 children).

A different pattern emerged as regards the length effect, which was more marked in the literacy students than in the two other groups. The Group by Length interaction, which was supported by the data in the ANOVA comparing the G3–4 children to the two BL subgroups, *BF*_inclusion_ = 4.72, reflected the fact literacy students showed a particularly strong performance drop on long items, which they read about 14% worse than short items (on the average: 72.92%, SD = 17.11, vs. 86.81%, SD = 14.45, respectively), whereas basic education students displayed a length effect of only about 5% (on the average: 88.56%, SD = 5.88, on long items vs. 93.46%, SD = 7.01, on short ones), similar to the 4.4% average length effect (SD = 7.86) displayed by the G3–4 children (cf. Appendix 1).Thus, on long items the literacy students displayed lower performance compared to both the basic education students, *BF*_10_ = 15.48, and the G3–4 children, *BF*_10_ = 10.29, with the two latter groups not differing from each other *BF*_10_ = 0.58.

No evidence for an interaction with Group was observed in the ANOVA considering Lexicality and comparing the G3-4 children to the two BL subgroups (see average scores in Appendix 4).

On the adults only, there was no evidence for a three-way interaction in the ANOVA on accuracy considering both Lexicality and Length in addition to Group (see average scores in Appendix 6). Yet the literacy students presented a 4.69% (SD = 6.47) word length effect that was not observed in the basic education students, who displayed an average word length effect of 0.25% (SD = 5.2). The literacy students also showed a larger length effect on pseudowords than the basic education students (on average, 32.29%, SD = 18.6 vs. 14.22%, SD = 13.75, respectively). Similarly, in the ANOVA on total reading time (see average scores in Appendix 6), there was no evidence for an interaction between Lexicality, Length and Group; there was even evidence in favor of the absence of that interaction, *BF*_exclusion_ = 3.23.

In the ANOVA on phoneme deletion (see average scores in Appendix 4), evidence supported a main effect of Group, *BF*_inclusion_ = 84.74, but there was evidence neither for an effect of Structure nor for a Structure X Group interaction. There was particularly strong evidence for a difference between the G3–4 children and the literacy students, *BF*_10_ = 895.22, who displayed average correct deletion scores of 97.68% (SD = 8.11) and 70.13% (SD = 23.58), respectively. Yet there was also good evidence for a difference between the G3-4 children and the basic education students, who performed on average at 88.41% (SD = 12.45), *BF*_10_ = 7.37. There was no evidence that the two BL subgroups differed from each other, *BF*_10_ = 1.54.

Yet the literacy students clearly underperformed the basic education students on auditory acronyms, *BF*_+0_ = 5.12, with correct scores of 41.25% (SD = 18.54) and 72.73% (SD = 25.2), respectively. In that task, they also presented a higher rate of orthographic errors than the basic education students, *BF*_−0_ = 13.97, on average 31.25% (SD = 19.76) and 9.09% (SD = 8.55), respectively.

Thus, although the two subgroups of BL adults were both roughly matched to the G3-4 children on regular word reading, they presented quite different literacy profiles.

### Comparisons between the reading-level matched subgroups of basic literacy adults and dyslexic children

Given the different literacy profiles of the two subgroups of BL adults, we further compared the dyslexic children to either the literacy students or the basic education students that had been matched to the dyslexic children on regular word reading. On the whole set of regular words, there was no evidence for a Group effect, *BF*_inclusion_ = 0.44. Both the literacy students and the basic education students were thus reading regular words at a similar level as the dyslexic children, with average performance of 87.3% (SD = 8.23), 92.66% (SD = 5.65), and 89.41% (SD = 6.21), respectively.

**Fig. 6:**
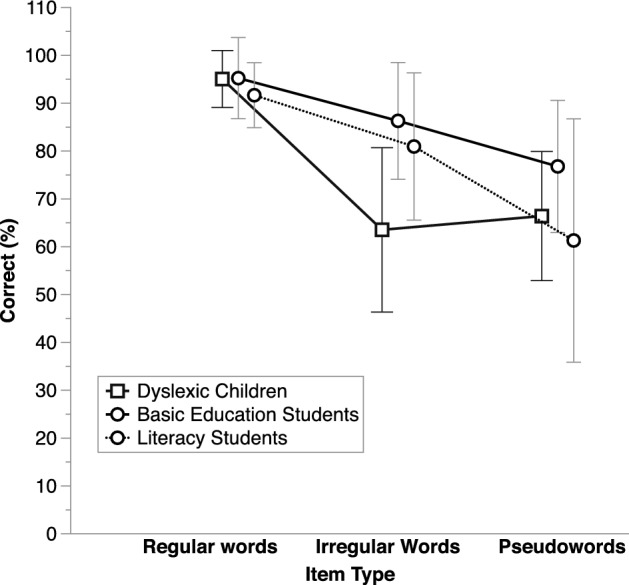
Average correct performance (%) on the reading tests, separately for each item type and in each subgroup of basic literacy adults (basic education vs. literacy students) as well as in the dyslexic children matched on regular word reading. Error bars show standard deviations

In the ANOVAs on the regular and irregular words that were matched on length and frequency (Fig. 6), there was very strong evidence for a Group by Regularity interaction, *BF*_inclusion_ = 110.65. Confirming the former analysis, both BL adult groups read regular words at the about the same level as the dyslexic children, *BF*_10_ = 0.66 for the comparison with the literacy students and = 0.40 for the comparison with the basic education students. They both tended to read irregular words better than the dyslexic children, but evidence for a Group effect on irregular word reading was substantial only when comparing the dyslexic children to the basic education students, *BF*_10_ = 8.77 (comparison with the literacy students: *BF*_10_ = 2.28).

No interaction with Group was observed in the ANOVA considering Lexicality in addition to Group (see average scores in Appendix 5). Yet there was some evidence for an interaction with Group in the ANOVA considering Length in addition to Group, *BF*_inclusion_ = 2.59. Although there was no evidence for local between-group comparisons, all *BF*_10_ < 2.4, on long items the literacy students tended to display lower average scores (69.44%, SD = 15.13) than both the basic education students (84.52%, SD = 6.59), and the dyslexic children (76.56%, SD = 10.29). As performance across groups was more homogeneous on short items, with average reading scores of 84.92% (SD = 7.02), 88.49% (SD = 8.7) and 82.99 (SD = 7.02), respectively, this means that the length effect varied across groups, *BF*_inclusion_ = 3.4. The literacy students presented a stronger length effect than the basic education students (on average, 15.48%, SD = 7.32 vs. 3.97%, SD = 3.53, respectively, *BF*_10_ = 13.14), and tended to present a stronger length effect than the dyslexic children (on average, 6.42%, SD = 8.6, *BF*_10_ = 2.65), which was not the case of the basic education students, *BF*_10_ = 0.48.

Despite the literacy students’ very poor deletion performance on CVC items (54% correct on average, SD = 46.15), in the ANOVA on deletion scores (see average scores in Appendix 5) there was no evidence for either a Group effect or a Group X Structure interaction. Yet there was evidence for a Group effect on auditory acronyms, *BF*_inclusion_ = 5.58. The literacy students displayed much worse performance than the dyslexic children, *BF*_10_ = 20.88, with average correct responses of 41.25% (SD = 18.54) vs. 83.33% (SD = 12.29), respectively. This was not the case of the basic education students, who performed in-between the two other groups, with 73.44% correct (SD = 28.58), and hence did not differ from the dyslexic children, *BF*_10_ = 0.6.

## Discussion

The main aim of the present study was to examine the hypothesis that, compared to reading-matched typically reading children, BL French-speaking adults struggle on phonologically demanding tasks but are relatively better on orthographic demanding tasks and therefore present a similar performance profile to dyslexic children. Contrary to previous studies, we directly compared BL adults to both dyslexic children and typically reading children in various reading and metaphonological tests. As in former studies (e.g., Greenberg et al., 1997, 2002), we adopted a reading-level match design and, in addition to comparing the BL adults to dyslexic children, we compared, on the one hand, the BL adults to G3-4 children matched on regular words reading and, on the other hand, dyslexic children to either chronological age or reading level controls matched on the same material.

Table 3 summarizes the most important findings. In agreement with former data collected on English speakers (e.g., Greenberg et al., 1997, 2002; Thompkins & Binder, 2003), when reading the BL adults seemed to rely more on orthographic representations than typically reading children. In the present study, they displayed better irregular word reading scores than the children from Grades 3 and 4 to whom they were matched on regular word reading. Nonetheless, contrary to what had been reported in previous work (in English: Greenberg et al., 1997; in French: Eme et al., 2014), they did not differ from their RL controls on pseudoword reading. This is surprising, as reliance on visual memory has been interpreted as a direct result of the poor BL adults’ decoding skills (Greenberg et al., 1997, 2002). Nonetheless, in agreement with the idea that phonological processing is poorly developed in BL adults as well as with former data (e.g., Eme, 2006; Eme et al., 2014; Greenberg et al., 1997; Read & Ruyter, 1985; Thompkins & Binder, 2003), the BL adults of the present study struggled on phoneme awareness, displaying poorer scores than their RL controls. In addition, as in former work on English speakers (Greenberg et al., 1997, 2002), the BL adults relied strongly on orthography in a demanding phonological task such as auditory acronyms.

**Table 3:** Summary of the main findings of the present study

		Observed performance		
		Dyslexic children versus RL controls	BL adults versus RL controls	BL adults versus dyslexic children
**Expected performance in BL adults and dyslexic children**				
	Pseudoword reading	**<**	≈	**≈**
Similar weakness in phonological processing				
	Phoneme awareness	≈	**<**	**≈ For basic education students (both tasks) and for literacy students on phoneme deletion**
				On auditory acronyms, literacy students < dyslexic children
Similar relative strength in orthographic processing				
	Irregular word reading	** > **	**> for basic education students**	**≈ for literacy students**
			Literacy students ≈ RL controls	Basic education students > dyslexic children

BL adults: basic literacy adults. RL controls were typical children matched to the target group on regular word reading, namely children from Grade 3 or 4 matched to the BL adults, and children from Grade 1 or 2 matched to the dyslexic children. In bold: results that are in agreement with the hypothesis

Thus, overall, the present results generalize to French the results previously observed in English as regards BL adults’ strong reliance on visual memory in irregular word reading and in demanding phonological tasks and corroborate Greenberg et al. (1997, 2002) idea that BL adults and typical children tend to use different approaches in both kinds of tasks, with the BL adults probably relying on compensatory strategies.

As BL adults, compared to controls matched on regular word reading, the dyslexic children were relatively better at reading irregular words. Yet, they also displayed poorer pseudoword reading than their RL controls, even though there was no evidence of poorer phoneme awareness. This was the mirror pattern of what was observed in the BL adults who, compared to their RL controls, were poorer at phoneme awareness but not at pseudoword reading.

Direct comparison of the dyslexic children and the BL adults who were matched on regular word reading showed that the latter displayed a marked advantage for irregular word reading. Remarkably, despite their superior reading performance, the BL adults did not outperform the dyslexic children on phoneme awareness and even tended to display poorer scores, at least on auditory acronyms. Their poor performance on this task was due to their numerous orthography-based errors, proportionally more numerous than in the typically reading children from Grades 5 and 6. Again, this suggests strong reliance on orthography when performing phonologically complex tasks. The orthography-based strategy of the BL adults correlated negatively with their reading performance.

The absence of difference between the dyslexic children’s and the BL adults’ deletion scores may seem at odds with the Spanish data reported by Jiménez et al. (2010), according to which BL adults performed worse than dyslexic children on phoneme awareness despite being matched for reading level. However, beyond the fact that these authors used a composite phoneme awareness score based on several tasks (phoneme blending, isolation, segmentation, and deletion), this discrepancy probably reflects two facts. First, as already commented on, the number of BL adults and dyslexic children matched on regular word reading was quite low in the present study and hence a null result should be taken with the utmost caution. Second, the BL adults of Jimenez et al.’s study were matched with 2nd graders in terms of reading accuracy, whereas the adult participants of the present study (at least the basic education students) were more advanced in the reading acquisition process. Results similar to those of Jimenez et al. would probably have been found if only literacy students had been involved in the present study.

This idea is supported by the different literacy profiles observed in the two subgroups of BL adults. The literacy students performed worse on phoneme awareness than the basic education students, at least in a challenging task such as auditory acronyms. In the latter task, only the literacy students, but not the basic literacy students, even performed worse than the dyslexic children to whom they had been matched on regular word reading.

The literacy students also presented a stronger length effect in reading than both the basic education students and the RL typical children and tended to present a stronger length effect than the dyslexic children, which was not the case of the basic education students.

Much on the contrary, the basic education students displayed a similar length effect as both their RL controls (G3–4 children) and the dyslexic children, while outperforming them on irregular word reading, which was not the case of the literacy students.

These results illustrate the need for future studies on larger groups of BL adults that should aim at better characterizing the difference between subgroups of BL adults, such as the literacy and basic education students examined in the present study.

In conclusion, the originality of our research lies in directly investigating the hypothesis that dyslexic children and BL adults present similar reading patterns. This hypothesis was only partly supported by the data, with results depending on the specific subgroup of BL adults considered. While the literacy students presented poorer phoneme awareness and a somewhat stronger length effect in reading than the dyslexic children, this was not the case of the basic education students. This seems to indicate that only the latter were comparable to dyslexic children in terms of phonological recoding processes. Yet, the reading pattern of the basic education students was not similar to that of the dyslexic children, as they largely outperformed them on irregular word reading. Thus, while similar to the dyslexic children on phonological recoding, the basic education students seem to have developed better orthographic representations. It would be interesting to check whether similar results would be found both on larger groups of BL adults and in children matched to their dyslexic peers in terms of phonological recoding, as assessed by phoneme awareness performance and strength of the length effect in reading. If this were the case, it would point to specific difficulties in constructing orthographic representations as a hallmark of developmental dyslexia.

Interestingly, the BL adults, and in particular the literacy students, seemed to rely frequently on orthography in performing a complex phoneme awareness task such as auditory acronyms. As we did not collect the proportion of orthographic errors made by the dyslexic children in this task, it remains to be investigated whether BL adults rely more on orthography than dyslexic children in such demanding metaphonological tasks.

### Educational perspectives

Future studies should aim at understanding whether the peculiar profiles presented by the BL adults, and in particular by the literacy adults, is intrinsic to adult literacy acquisition, or whether it is related to the fact that they are taught and trained to read and write in a different way compared to both typical and dyslexic children. All the dyslexic children examined in the present study benefited from remediation sessions and hence were probably trained on phonological awareness exercises. As regards typical children, whereas many of their teachers have the notion that acquiring metaphonological awareness, especially at the level of the phoneme, is useful or even necessary, this is often not the case in adult literacy classes. The reasons for this are probably multiple, among which the endorsement of one or several of the following beliefs: (1) that the orthographic knowledge displayed by the BL adults presupposes already fair metaphonological awareness skills (moreover, most of the adults’ teachers do not distinguish clearly phoneme awareness from phonological awareness); (2) that “playing” with speech sounds and phonemes would not be well accepted and understood as crucial by the BL adults themselves; and (3) that the time devoted to phonological and phoneme awareness training would reduce considerably the time available for the two most urgent learnings instrumental for success in the official exams, namely fluent text reading comprehension and autonomy (including correct orthography) in text writing. It will be important to check whether less time is devoted to phonological awareness training in adult literacy classes compared to what is done in children’s ones and regarding the time devoted to reading, writing, and text comprehension activities. This would allow establishing whether skipping the more basic foundational processes of developing phoneme awareness and, more generally, phonological awareness, is or not detrimental to the final operational objectives.

It would be equally important to examine larger samples of BL adults to check what variables may help distinguishing between literacy students and basic education students, beyond their literacy level per se. This would imply investigating issues such as what instruction did the BL adults receive as children and how long they have been trained as adults. Better understanding all these characteristics may have strong implications for adult educational policy, as not all countries organize distinct adult literacy and adult basic education classes.

## Data Availability

The data that support the findings of this study are openly available in the Open Science Framework at https://osf.io/a3rku/.

## Appendix

### Appendix 1: Results of the Bayesian ANOVAs for the comparisons between the basic literacy adults and the typically reading children matched on the whole set of non-irregular words

**Table:**

Regularity in reading: reading—regular versus irregular words					
Models comparison	P(M)	P(M|data)	BF_M_	BF_10_	Error%
Null model (incl. subject)	0.2	3.527E−16	1.411E−15	1	
Regularity + group + regularity * group	0.2	0.998	2246964	2.83E+15	3.064
Regularity + group	0.2	0.001	0.006	3.941E+12	1.351
Regularity	0.2	3.871E−4	0.002	1.097E+12	2.1
Group	0.2	5.716E−16	2.286E−15	1621	1.775

Analysis of effects	P(incl)	P(excl)	P(incl|data)	P(excl|data)	BF _incl_
Regularity	0.4	0.4	0.002	9.243E−16	**1.92E+12**
Group	0.4	0.4	0.001	3.871E−4	**3.591**
Regularity * group	0.2	0.2	0.998	0.001	**718.171**

Lexicality in reading: words versus pseudowords					
Model comparison	P(M)	P(M|data)	BF_M_	BF_10_	Error%
Null model (incl. subject)	0.2	2.314E−12	9.255E−12	1	
Lexicality	0.2	0.577	5.448	2.492E+11	0.872
Lexicality + group + lexicality * group	0.2	0.228	1.182	9.858E+10	2.425
Lexicality + group	0.2	0.195	0.971	8.44E+10	1.071
Group	0.2	5.997E−13	2.399E−12	0.259	2.416

Analysis of effects	P(incl)	P(excl)	P(incl|data)	P(excl|data)	BF_incl_
Lexicality	0.4	0.4	0.772	2.913E−12	**2.65E+11**
Group	0.4	0.4	0.195	0.577	0.339
Lexicality * group	0.2	0.2	0.228	0.195	1.168

Item length in reading: short versus long items (words and pseudowords)					
Model comparison	P(M)	P(M|data)	BF_M_	BF_10_	Error %
Null model (incl. subject)	0.2	5.014E−6	2.006E−5	1	
Length	0.2	0.595	5.885	118722.85	0.983
Length + group	0.2	0.212	1.079	42364.486	2.45
Length + group + length * group	0.2	0.192	0.952	38333.17	2792
Group	0.2	1.534E−6	6.137E−6	0.306	0.681

Analysis of effects	P(incl)	P(excl)	P(incl|data)	P(excl|data)	BF_incl_
*Length*	0.4	0.4	0.808	6.549E−6	**123348.869**
Group	0.4	0.4	0.212	0.595	0.357
Length * group	0.2	0.2	0.192	0.212	0.905

Descriptives: Length	Group	N	Mean (%)	SD
Short	Basic literate adults	25	91.33	10.19
	G3–4 children	41	89.84	5.58
Long	Basic literate adults	25	83.56	12.8
	G3–4 children	41	85.43	9

Phoneme deletion: items with simple (CVC) versus complex (CCV) structure					
Model comparison	P(M)	P(M|data)	BF_M_	BF_10_	Error%
Structure + group	0.2	0.448	3.241	1	
Group	0.2	0.35	2.151	0.781	1.771
Structure + group + structure * group	0.2	0.173	0.834	0.385	2.841
Structure	0.2	0.016	0.066	0.036	2.206
Null model (incl. subject)	0.2	0.014	0.057	0.031	1.292

Analysis of effects	P(incl)	P(excl)	P(incl|data)	P(excl|data)	BF_incl_
Structure	0.4	0.4	0.464	0.364	1.275
Group	0.4	0.4	0.797	0.03	**26399**
Structure * group	0.2	0.2	0.173	0.448	0.385

All models include subject. Effects supported by substantial, strong or very strong evidence are in bold.

### Appendix 2: Results of the Bayesian ANOVAs for comparisons between the dyslexic children and reading-level or chronological age matched typically reading children

**Table:**

Regularity in reading: reading—regular versus irregular words					
Model comparison	P(M)	P(M|data)	BF_M_	BF_10_	Error%
Null model (incl. subject)	0.2	1.769E−63	7.078E−63	1	
Regularity + group + regularity * group	0.2	1	1.715E+20	5.651E+62	2.551
Regularity + group	0.2	2.332E−20	9.33E−20	1.318E+43	2.767
Regularity	0.2	1.361E−39	5.446E−39	7.694E+23	0.894
Group	0.2	2.012E−46	8.048E−46	1.137E+17	2.974

Analysis of effects	P(incl)	P(excl)	P(incl|data)	P(excl|data)	BF_incl_
Regularity	0.4	0.4	2.332E−20	2.012E−46	**1.159E+26**
Group	0.4	0.4	2.332E−20	1.361E−39	**1.713E+19**
Regularity * group	0.2	0.2	1	2.332E−20	**4.287E+19**

Lexicality in reading: words versus pseudowords					
Model comparison	P(M)	P(M|data)	BF_M_	BF_10_	Error%
Null model (incl. subject)	0.2	1.927E−43	7.887E−43	1	
Lexicality + group + lexicality * group	0.2	0.999	7692.531	5.069E+42	6.622
Lexicality + group	0.2	5.197E−4	0.002	2.636E+39	1.544
Lexicality	0.2	4.375E−20	1.75E−19	2.219E+23	0.906
Group	0.2	2.691E−27	1.076E−26	1.365E+16	0.683

Analysis of effects	P(incl)	P(excl)	P(incl|data)	P(excl|data)	BF_incl_
Lexicality	0.4	0.4	5.197E−4	2.691E−27	**1.932E+23**
Group	0.4	0.4	5.197E−4	4.375E−20	**1.188E+16**
Lexicality * group	0.2	0.2	0.999	5.197E−4	**1923133**

Item length in reading: short versus long items (words and pseudowords)					
Model comparison	P(M)	P(M|data)	BF_M_	BF_10_	Error %
Null model (incl. subject)	0.2	5.127E−25	2.051E−24	1	
Length + group + length * group	0.2	0.656	7.630	1.28E+24	2.119
Length + group	0.2	0.344	2.097	6.708E+23	2.726
Group	0.2	1.782E−9	7.127E−9	3.475E+15	1.333
Length	0.2	1.147E−16	4.59E−16	2.238E+8	2.605

Analysis of effects	P(incl)	P(excl)	P(incl|data)	P(excl|data)	BF_incl_
Length	0.4	0.4	0.344	1.782E−9	1.93E+8
Group	0.4	0.4	0.344	1.147E−16	2.997E+15
Length * group	0.2	0.2	0.656	0.344	1.908

Descriptives: Length	Group	N	Mean (%)	SD
Short	Dyslexic children	53	74.48	14.13
	G1-2 children	26	80.02	10.74
	G5-6 children	47	96.28	3.29
Long	Dyslexic children	53	64.94	16.58
	G1-2 children	26	71.47	21.67
	G5-6 children	47	92.55	4.85

Phoneme deletion: items with simple (CVC) versus complex (CCV) structure					
Model comparison	P(M)	P(M|data)	BF_M_	BF_10_	Error %
Structure + group	0.2	0.459	3397	1	
Structure + group + structure * group	0.2	0.333	1996	0.725	2.313
Group	0.2	0.208	1049	0.453	1.782
Structure	0.2	3.764E−5	1.506E−4	8.197E−5	1.995
Null model (incl. subject)	0.2	1.911E−5	7.645E−5	4.162E−5	1.673

Analysis of effects	P(incl)	P(excl)	P(incl|data)	P(excl|data)	BF_incl_
Structure	0.4	0.4	0.459	0.208	2.21
Group	0.4	0.4	0.667	5.675E−5	**11752.859**
Structure * group	0.2	0.2	0.333	0.459	0.725

All models include subject. Effects supported by substantial, strong or very strong evidence are in bold.

### Appendix 3: Results of the Bayesian ANOVAs for the comparisons between the basic literacy adults and the dyslexic children matched on the whole set of non-irregular words

**Table:**

Regularity in reading: reading: regular versus irregular words					
Model comparison	P(M)	P(M|data)	BF _M_	BF_10_	Error %
Null model (incl. subject)	0.2	9.493E−10	3.797E−9	1	
Regularity + group + regularity * group	0.2	0.994	684.998	1.047E+9	1608
Regularity + group	0.2	0.004	0.017	4.331E+6	1666
Regularity	0.2	0.002	0.007	1.784E+6	1555
Group	0.2	1.102E−9	4.407E−9	1161	1988

Analysis of effects	P(incl)	P(excl)	P(incl|data)	P(excl|data)	BF_incl_
Regularity	0.4	0.4	0.006	2.051E−9	**2.831E+6**
Group	0.4	0.4	0.004	0.002	2427
Regularity * group	0.2	0.2	0.994	0.004	**241.796**

Lexicality in reading: words versus pseudowords					
Model comparison	P(M)	P(M|data)	BF_M_	BF_10_	Error %
Null model (incl. subject)	0.2	1.053E−7	4.211E−7	1	
Lexicality	0.2	0.633	6.888	6.01E+6	1.33
Lexicality + group	0.2	0.277	1.533	2.632E+6	2.025
Lexicality + group + lexicality * group	0.2	0.09	0.397	858540.364	1.684
Group	0.2	3.562e −8	1.425e −7	0.338	1.408

Analysis of effects	P(incl)	P(excl)	P(incl|data)	P(excl|data)	BF_incl_
Lexicality	0.4	0.4	0.91	1.409E−7	**6.457E+6**
Group	0.4	0.4	0.277	0.633	0.438
Lexicality * group	0.2	0.2	0.09	0.277	0.326

Item length in reading: short versus long items (words and pseudowords)					
Model comparison	P(M)	P(M|data)	BF_M_	BF_10_	Error %
Null model (incl. subject)	0.2	5.787E−4	0.002	1	
Length	0.2	0.57	5.31	985.574	2.329
Length + group	0.2	0.284	1.584	490.086	2.254
Length + Group + length * group	0.2	0.145	0.68	250.942	2.293
Group	0.2	2.522E−4	0.001	0.436	0.591

Analysis of effects	P(incl)	P(excl)	P(incl|data)	P(excl|data)	BF_incl_
Length	0.4	0.4	0.854	8.309E−4	**1027.714**
Group	0.4	0.4	0.284	0.571	0.497
Length * Group	0.2	0.2	0.145	0.284	0.512

Descriptives: length	Group	N	Mean (%)	SD
Short	Dyslexic children	16	82.99	7.02
	Basic literacy adults	14	86.7&	11.64
Long	Dyslexic children	16	76.56	10.29
	Basic literacy adults	14	76.98	13.67

Phoneme deletion: items with simple (CVC) versus complex (CCV) structure					
Model comparison	P(M)	P(M|data)	BF_M_	BF_10_	Error%
Structure	0.2	0.373	2.376	1	
Null model (incl. subject)	0.2	0.228	1.181	0.612	1.338
Structure + group	0.2	0.197	0.979	0.528	2.002
Group	0.2	0.122	0.557	0.328	1.572
Structure + group + structure * group	0.2	0.08	0.35	0.216	2.696

Analysis of effects	P(incl)	P(excl)	P(incl|data)	P(excl|data)	BF _incl_
Structure	0.4	0.4	0.569	0.35	1.626
Group	0.4	0.4	0.319	0.601	0.531
Structure * group	0.2	0.2	0.08	0.197	0.409

All models include subject. Effects supported by substantial, strong or very strong evidence are in bold.

### Appendix 4: Results of the Bayesian ANOVAs for the comparisons between the two groups of basic literacy adults and the typically reading children matched on the whole set of non-irregular words

**Table:**

Regularity in reading: reading—regular versus irregular words					
Model comparison	P(M)	P(M|data)	BF_M_	BF_10_	Error %
Regularity + group + regularity * group	0.2	0.998	1913.986	1	
regularity + group	0.2	0.002	0.008	0.002	3.52
Regularity	0.2	1.888E−4	7.554E−4	1.892E−4	2.538
Group	0.2	4.619E−16	1.848E−15	4.629E−16	1.708
Null model (incl. subject)	0.2	1.737E−16	6.946E−16	1.74E−16	1.574

Analysis of effects	P(incl)	P(excl)	P(incl|data)	P(excl|data)	BF_incl_
Regularity	0.4	0.4	0.002	6.356E−16	**3.281E+12**
Group	0.4	0.4	0.002	1.888E−4	**10.045**
Regularity * group	0.2	0.2	0.998	0.002	**526.131**

Lexicality in reading: words versus pseudowords					
Model comparison	P(M)	P(M|data)	BF_M_	BF_10_	Error%
Lexicality + group + lexicality * group	0.2	0.455	3336	1	
Lexicality + group	0.2	0.446	3221	0.981	4.268
Lexicality	0.2	0.099	0.44	0.218	1.616
Group	0.2	1.09E−12	4.361E−12	2.3971E−12	1.411
Null model (incl. subject)	0.2	3.952E−13	1.581E−12	8.691E−13	1.282

Analysis of effects	P(incl)	P(excl)	P(incl|data)	P(excl|data)	BF_incl_
Lexicality	0.4	0.4	0.545	1.486E−12	**3.67E+11**
Group	0.4	0.4	0.446	0.099	**4.5**
Lexicality * group	0.2	0.2	0.455	0.446	1.019

Descriptives : lexicality	Group	N	Mean (%)	SD
Words	G3-4 Children	41	91.16	5.66
	Basic education students	17	95.47	4.56
	Literacy students	8	86.72	9.77
Pseudowords	G3-4 children	41	80.59	10.65
	Basic education students	17	82.11	10.18
	Literacy students	8	66.15	27.23

Item length in reading: short versus long items (words and pseudowords)					
Model comparison	P(M)	P(M|data)	BF_M_	BF_10_	Error%
Length + group + length * group	0.2	0.801	16.143	1	
Length + group	0.2	0.17	0.817	0.212	11.473
Length	0.2	0.029	0.119	0.036	6.606
Group	0.2	1.315E−6	5.259E−6	1.641E−6	6.559
Null model (incl. subject)	0.2	2.425E−7	9.701E−7	3.026E−7	6.477

Analysis of effects	P(incl)	P(excl)	P(incl|data)	P(excl|data)	BF_incl_
Length	0.4	0.4	0.199	1.557E−6	**127507.398**
Group	0.4	0.4	0.17	0.029	**5.864**
Length * group	0.2	0.2	0.801	0.17	**4.724**

Phoneme deletion: items with simple (CVC) versus complex (CCV) structure					
Model comparison	P(M)	P(M|data)	BF_M_	BF_10_	Error %
Structure + group + structure * group	0.2	0.531	4.527	1	
Structure + group	0.2	0.268	1.462	0.504	3.936
Group	0.2	0.196	0.975	0.369	1.533
Structure	0.2	0.003	0.012	0.006	13.022
Null model (incl. subject)	0.2	0.002	0.009	0.004	1.418

Analysis of effects	P(incl)	P(excl)	P(incl|data)	P(excl|data)	BF_incl_
Structure	0.4	0.4	0.271	0.198	1.365
Group	0.4	0.4	0.464	0.005	**84.74**
Structure * group	0.2	0.2	0.531	0.268	1.984

Descriptive : structure	Group	N	Mean (%)	SD
CVC items	G3-4 children	28	100	0
	Basic education students	11	88.64	11.46
	Literacy students	5	86.25	12.02
CCV items	G3-4 children	28	95.36	16.21
	Basic education students	11	88.18	19.91
	Literacy students	5	54	46.15

All models include subject. Effects supported by substantial, strong or very strong evidence are in bold.

### Appendix 5: Results of the Bayesian ANOVAs for the comparisons between the two groups of basic literacy adults and the dyslexic children matched on the whole set of non-irregular words

**Table:**

Regularity in reading: reading—regular versus irregular words					
Model comparison	P(M)	P(M|data)	BF_M_	BF_10_	Error%
Regularity + group + regularity * group	0.2	0.985	258.196	1	
Regularity + group	0.2	0.009	0.036	0.009	16.95
Regularity	0.2	0.006	0.026	0.006	4.427
Null model (incl. subject)	0.2	3.4E−9	1.36E−8	3.453E−9	1.317
Group	0.2	2.297E−9	9.189E−9	2.333E−9	1.404

Analysis of effects	P(incl)	P(excl)	P(incl|data)	P(excl|data)	BF_incl_
Regularity	0.4	0.4	0.015	5.697E−9	**2.678E+6**
Group	0.4	0.4	0.009	0.006	1.4
Regularity * group	0.2	0.2	0.985	0.009	**110.653**

Lexicality in reading: words versus pseudowords					
Model comparison	P(M)	P(M|data)	BF_M_	BF_10_	Error%
Lexicality	0.2	0.513	4.222	1	
Lexicality + group	0.2	0.361	2.265	0.704	27.77
Lexicality + group + lexicality * group	0.2	0.125	0.572	0.244	1.755
Null model (incl. subject)	0.2	8.619E−8	3.448E−7	1.679E−7	0.951
Group	0.2	3.677E−8	1.471E−7	7.161E−8	1.19

Analysis of effects	P(incl)	P(excl)	P(incl|data)	P(excl|data)	BF_incl_
Lexicality	0.4	0.4	0.875	1.23E−7	**7.116E+6**
Group	0.4	0.4	0.361	0.513	0.704
Lexicality * group	0.2	0.2	0.125	0.361	0.346

Descriptives: Lexicality	Group	N	Mean (%)	SD
Words	Dyslexic children	16	86.59	7.22
	Basic education students	7	91.37	4.57
	Literacy students	7	85.12	9.35
Pseudowords	Dyslexic children	16	66.41	13.48
	Basic education students	7	76.79	13.79
	Literacy students	7	61.31	25.43

Item length in reading: short versus long items (words and pseudowords)					
Model comparison	P(M)	P(M|data)	BF_M_	BF_10_	Error %
Length + group + length * group	0.2	0.53	4.516	1	
Length	0.2	0.265	1.439	0.499	4.091
Length + group	0.2	0.205	1.03	0.386	4.665
Null model (incl. subject)	0.2	2.685E−4	0.001	5.064E−4	3.768
Group	0.2	1.901E−4	7.606E−4	3.585E−4	3.896

Analysis of effects	P(incl)	P(excl)	P(incl|data)	P(excl|data)	BF _incl_
Length	0.4	0.4	0.469	4.587E−4	**1023.113**
Group	0.4	0.4	0.205	0.265	0.774
Length * group	0.2	0.2	0.53	0.205	2.591

Phoneme deletion: items with simple (CVC) versus complex (CCV) structure					
Model comparison	P(M)	P(M|data)	BF_M_	BF_10_	Error %
Structure	0.2	0.307	1.772	1	
Structure + group	0.2	0.209	1.055	0.68	1.958
Null model (incl. subject)	0.2	0.185	0.91	0.604	1.553
Structure + group + structure * group	0.2	0.177	0.862	0.577	2.363
Group	0.2	0.122	0.554	0.396	1.776

Analysis of effects	P(incl)	P(excl)	P(incl|data)	P(excl|data)	BF_incl_
Structure	0.4	0.4	0.516	0.307	1.68
Group	0.4	0.4	0.33	0.492	0.671
Structure * group	0.2	0.2	0.177	0.209	0.85

Descriptives: structure	Group	N	Mean (%)	SD
CVC items	Dyslexic children	11	96.02	5.06
	Basic education students	4	87.5	16.93
	Literacy students	5	86.25	12.02
CCV items	Dyslexic children	11	81.82	30.27
	Basic education students	4	95	5.77
	Literacy students	5	54	46.15

All models include subject. Effects supported by substantial, strong or very strong evidence are in bold.

### Appendix 6: Results of the Bayesian ANOVAs examining the effects of lexicality and length in reading in the two groups of basic literacy adults

**Table:**

Lexicality and length in reading: reading accuracy					
Model comparison	P(M)	P(M|data)	BF_M_	BF_10_	Error %
Lexicality + length + group + lexicality * length + lexicality * group + length * group	0.053	0.319	8.44	1	
Lexicality + length + group + lexicality * length + length * group	0.053	0.304	7.879	0.954	22.52
Lexicality + length + group + lexicality * length + lexicality * group + length * group + lexicality * length * group	0.053	0.202	4.554	0.633	22.589
Lexicality + length + group + lexicality * length	0.053	0.086	1.693	0.269	22.468
Lexicality + length + group + lexicality * length + lexicality * group	0.053	0.062	1.189	0.194	22.808
Lexicality + length + lexicality * length	0.053	0.026	0.476	0.081	22.461
Lexicality + length + group + length * group	0.053	2.806E−4	0.005	8.79E−4	22.484
Lexicality + length + group + lexicality * group + length * group	0.053	1.853E−4	0.003	5.805E−4	22.455
Lexicality + length + group	0.053	1.283E−4	0.002	4.019E−4	22.456
Lexicality + length + group + lexicality * group	0.053	8.748E−5	0.002	2.741E−4	22.684
Lexicality + length	0.053	4.281E−5	7.706E−4	1.341E−4	22.368
Lexicality + group	0.053	8.803E−8	1.585e−6	2.758E−7	22.546
Lexicality + group + lexicality * group	0.053	4.374E−8	7.874E−7	1.37E−7	22.4
Lexicality	0.053	2.921E−8	5.259E−7	9.152E−8	22.353
Length + group	0.053	2.72E−11	4.895E−10	8.52E−11	22.412
Length + group + length * group	0.053	2.649E−11	4.769E−10	8.3E−11	22.436
Length	0.053	1.005E−11	1.808E−10	3.147E−11	22.386
Group	0.053	3.314E−13	5.965E−12	1.038E−12	22.347
Null model (incl. subject)	0.053	1.371E−13	2.468E−12	4.295E−13	22.332

Analysis of effects	P(incl)	P(excl)	P(incl|data)	P(excl|data)	BF_incl_
Lexicality	0.263	0.263	4.518E−4	6.421E−11	**7.037E+6**
Length	0.263	0.263	2.586E−4	1.61E−7	**1606.113**
Lexicality * length	0.263	0.263	0.797	7.245E−4	**1100.621**
Group	0.263	0.263	0.086	0.026	**3.334**
Lexicality * group	0.263	0.263	0.381	0.391	0.976
Length * group	0.263	0.263	0.624	0.148	**4.213**
Lexicality * length * group	0.053	0.053	0.202	0.319	0.633

Descriptives: lexicality	Length	Group	N	Mean (%)	SD
Words	Short	Basic education students	17	95.59	5.79
		Literacy students	8	89.06	10.9
	Long	Basic education students	17	95.34	4.63
		Literacy students	8	84.38	9.64
PM	Short	Basic education students	17	89.22	11.32
		Literacy Students	8	82.29	22.47
	Long	Basic education students	17	75	13.18
		Literacy students	8	50	33.92

Lexicality and length in reading: total reading time					
Model comparison	P(M)	P(M|data)	BF_M_	BF_10_	Error %
Lexicality + length + group + lexicality * length + length * group	0.053	0.497	17.761	1	
Lexicality + length + group + lexicality * length + lexicality * group + length * group	0.053	0.210	4.790	0.423	12.835
Lexicality + length + group + lexicality * length	0.053	0.103	2.062	0.207	10.105
Lexicality + length + lexicality * length	0.053	0.089	1.751	0.178	3.931
Lexicality + length + group + lexicality * length + lexicality * group + length * group + lexicality * length * group	0.053	0.065	1.257	0.131	5.854
Lexicality + length + group + lexicality * length + lexicality * group	0.053	0.036	0.681	0.073	10.867
Lexicality + length + group + length * group	0.053	5.359E−11	9.646E−10	1.079E−10	4.965
Lexicality + length + group	0.053	4.354E−11	7.838E−10	8.767E−11	4.817
Lexicality + length	0.053	4.347E−11	7.825E−10	8.753E−11	4.19
Lexicality + length + group + lexicality * group + length * group	0.053	1.594E−11	2.869E−10	3.209E−11	4.38
Lexicality + length + group + lexicality * group	0.053	1.573E−11	2.832E−10	3.168E−11	13.889
Length	0.053	4.167E−19	7.5E−18	8.39E−19	3.355
Length + group	0.053	3.67E−19	6.606E−18	7.389E−19	3.624
Length + group + length * group	0.053	2.277E−19	4.098E−18	4.584E−19	3.689
Lexicality	0.053	1.888E−26	3.398E−25	3.801E−26	3.397
Lexicality + group	0.053	1.371E−26	2.468E−25	2.76E−26	4.065
Lexicality + group + lexicality * group	0.053	3.948E−27	7.106E−26	7.949E−27	3.63
Null model (incl. subject)	0.053	1.118E−29	2.012E−28	2.25E−29	3.219
Group	0.053	7.069E−30	1.272E−28	1.423E−29	3.53

Analysis of effects	P(incl)	P(excl)	P(incl|data)	P(excl|data)	BF_incl_
Lexicality	0.263	0.263	1.406E−10	1.011E−18	**1.39E+8**
Length	0.263	0.263	1.027E−10	3.665E−26	**2.811E+15**
Lexicality * length	0.263	0.263	0.935	1.723E−10	**5.426E+9**
Group	0.263	0.263	0.103	0.089	1.16
Lexicality * group	0.263	0.263	0.247	0.599	0.411
Length * group	0.263	0.263	0.707	0.139	**5.076**
Lexicality * length * group	0.053	0.053	0.065	0.21	0.31

Descriptive: lexicality	Length	Group	N	Mean (s)	SD
Words	Short	Basic education students	17	4.58	2.79
		Literacy students	8	5.02	0.82
	Long	Basic education students	17	6.37	2.21
		Literacy students	8	8.78	2.09
Pseudo	Short	Basic education students	17	5.06	2.2
		Literacy students	8	6.13	2.15
	Long	Basic education students	17	12.29	3.92
		Literacy students	8	14.95	2.89

All models include subject. Effects supported by substantial, strong or very strong evidence are in bold.

## Footnotes

1 Stimuli were two to four phonemes monosyllabic words in the phoneme counting task; chance level might therefore be considered as being 33%.

2 Testing had to be interrupted because of school closures during the Covid-19 pandemic, which did not allow time to collect all metaphonological data on all participants. Once the health situation improved, we could not test them because either they were in higher grades or adults had stopped their education.

3 Those values were called *Baws factor* by Sebastiaan Mathôt, but for sake of coherence, we kept the JASP terminology, namely *BF*_inclusion_. When looking at evidence in favor of H_1_, BF_inclusion_ indicates the change from prior to posterior inclusion odds.

4 Due to the administration guidelines of the BELEC, we could not separate performance on short vs. long words and short versus long pseudowords. Yet for the BL adults we recorded separately the data on these items.

5 *BF*_exclusion_ is the equivalent to *BF*_inclusion_ when looking at evidence in favor of H_0_.

6 The IES is calculated by dividing response time by the proportion of correct responses. In the present case, since total reading times are expressed in s and divided by proportions, IES is expressed in s as well.
